# D-Serine May Ameliorate Hippocampal Synaptic Plasticity Impairment Induced by Patients’ Anti-N-methyl-D-aspartate Receptor Antibodies in Mice

**DOI:** 10.3390/biomedicines12122882

**Published:** 2024-12-18

**Authors:** Hanyu Luo, Xiaoyue Yang, Jiaxin Yang, Ziyao Han, Dishu Huang, Jianxiong Gui, Ran Ding, Hengsheng Chen, Li Cheng, Jiannan Ma, Li Jiang

**Affiliations:** Department of Neurology, Children’s Hospital of Chongqing Medical University, National Clinical Research Center for Child Health and Disorders, Ministry of Education Key Laboratory of Child Development and Disorders, Chongqing Key Laboratory of Child Neurodevelopment and Cognitive Disorders, Chongqing 400014, China

**Keywords:** anti-N-methyl-D-aspartate receptor encephalitis, D-serine, synaptic plasticity, mice model, anti-NMDAR antibody

## Abstract

**Objective**: To establish a mouse model of anti-N-methyl-D-aspartate receptor (NMDAR) encephalitis and assess the potential therapeutic benefits of D-serine supplementation in mitigating synaptic plasticity impairments induced by anti-NMDAR antibodies. **Methods**: Anti-NMDAR antibodies were purified from cerebrospinal fluid (CSF) samples of patients diagnosed with anti-NMDAR encephalitis and verified using a cell-based assay. CSF from patients with non-inflammatory neurological diseases served as the control. These antibodies were then injected intraventricularly into C57BL/6 mice. Forty-eight hours following the injection, mice were administered either D-serine (500 mg/kg) or sterile saline intraperitoneally for three consecutive days. Subsequent analyses included Western blotting, immunofluorescence, electrophysiological studies, and a series of behavioral tests to assess pathological changes caused by anti-NMDAR antibodies. **Results**: Mice injected with anti-NMDAR antibodies exhibited a significant reduction in hippocampal long-term potentiation compared to controls, which was notably ameliorated by D-serine treatment. Additionally, these mice displayed decreased levels of hippocampal membrane NMDAR1 protein and postsynaptic NMDAR1 density. However, D-serine administration did not significantly alter these conditions. Notably, no significant behavioral differences were observed between mice injected with anti-NMDAR antibodies and controls in open fields, elevated plus maze, novel object recognition, or Morris water maze tests. **Conclusions**: Our findings indicate that exogenous D-serine can improve hippocampal plasticity impairments caused by anti-NMDAR antibodies but does not reverse the decreased expression of NMDAR. Furthermore, a single intraventricular injection of patients’ antibodies was insufficient to induce anti-NMDAR encephalitis-related behaviors in mice.

## 1. Introduction

Anti-N-methyl-D-aspartate receptor (NMDAR) encephalitis is the most common autoimmune encephalitis, with the first case reported in 2007. During the acute stage, the disease is mainly characterized by psychiatric disorders, seizures, movement disorders, speech dysfunction, autonomic dysfunction, and central hypoventilation [1]. Immunotherapy, including intravenous immunoglobulin, plasma exchange, steroids, and other second-line treatments, is widely recommended by current guidelines [2]. However, many patients still have bad outcomes despite receiving standard immunotherapy [3]. Recent studies indicated that, unlike acute neurological deficits, cognitive impairment is the primary factor affecting the quality of life of patients with anti-NMDAR encephalitis [4,5,6]. Therefore, in addition to current immunotherapy, further exploration of treatment that can improve cognitive function is crucial to improve the prognosis of patients with anti-NMDAR encephalitis.

Within the postsynaptic compartment, NMDAR is stabilized and anchored through the interaction between the extracellular domain of the GluN1 subunit and EphB2 receptors, which occurs due to the electrostatic interaction between the negatively charged GluN1 extracellular domain and the positively charged EphB2 under physiological conditions [7]. Previous studies have shown that anti-NMDAR antibodies specifically target the GluN1 subunit on neuronal membranes, disrupting the NMDAR-EphB2 interaction. This disruption leads to the lateral dispersion of NMDARs, followed by crosslinking of NMDARs by the antibodies, which triggers receptor internalization and ultimately results in a reduction in receptor clusters on the postsynaptic surface [8]. As NMDAR plays a crucial role in maintaining normal synaptic plasticity, hippocampal synaptic plasticity serves as the foundation for cognitive function. Thus, a leading hypothesis for anti-NMDAR encephalitis-related cognitive impairment could be sustained NMDAR hypofunction due to severe immune reaction or other underlying reasons. To support this hypothesis, Warikoo et al. initially conducted an in vitro study using rat hippocampal neurons. The results suggested that co-application of SGE-301, a positive allosteric modulator of NMDAR, could restore NMDAR hypofunction induced by patients’ CSF [9]. Further studies by Mannara et al. [10] and Radosevic et al. [11] demonstrated that SGE-301 could improve cognitive impairment caused by anti-NMDAR antibodies in mice models. These findings highlight the potential of NMDA receptor-modulating treatments as a promising adjunctive therapy beyond immunotherapy for anti-NMDAR encephalitis.

In addition to glutamate, the activation of NMDAR requires the binding of a co-agonist at the glycine binding site. D-serine, which binds to the glycine binding site with significantly higher affinity than glycine itself, is the primary endogenous co-agonist of NMDAR in the brain [12]. Both preclinical and clinical studies have demonstrated that a reduction in D-serine or defects in its key synthetic enzymes lead to significant impairments in synaptic plasticity and cognitive function [13,14]. Furthermore, exogenous D-serine supplementation has been shown to improve cognitive disorders associated with schizophrenia, depression, chronic lead exposure, Alzheimer’s disease, traumatic brain injury, and acute stress, as well as other related neuropsychiatric disorders [15,16,17,18]. For example, current evidence supports the deleterious role of NMDAR hypofunction in cognitive impairment associated with schizophrenia. Previous studies have demonstrated that D-serine supplementation successfully improves synaptic plasticity and memory by enhancing NMDAR activity in rodent models of schizophrenia [19,20]. Furthermore, clinical trials have reported significant improvements in cognitive function in patients with schizophrenia following D-serine treatment [21,22]. Psychosis is also a key symptom of anti-NMDAR encephalitis, and anti-NMDAR antibodies have been detected in a small group of patients with schizophrenia [23,24], suggesting potential similarities in the pathological changes between anti-NMDAR encephalitis and schizophrenia. Therefore, based on the theoretical foundation of NMDAR hypofunction induced by anti-NMDAR antibodies, it is reasonable to investigate whether the application of D-serine could improve NMDAR hypofunction in anti-NMDAR encephalitis and, consequently, enhance cognitive function.

Currently, the development of animal models for anti-NMDAR encephalitis encompasses both passive transfer and active immunization techniques. The mainstream method is the continuous infusion of patient-derived anti-NMDAR antibodies into the lateral ventricle using osmotic pumps for 14 days [25]. However, this approach requires substantial patient sample support. Several studies have attempted direct injection of patients’ antibodies into the hippocampus [26,27]. However, the substantial damage caused by this approach might affect the accuracy of short-term experiments. In this study, we first aim to ascertain whether a more straightforward method, a single direct injection of patient-derived anti-NMDAR antibodies into the lateral ventricle, could effectively establish an animal model. We then investigate whether D-serine, an endogenous co-agonist of NMDAR rather than an exogenous positive allosteric modulator, can ameliorate hippocampal synaptic plasticity impairments and cognitive deficits in mice for the first time.

## 2. Materials and Methods

### 2.1. Patient and Anti-NMDAR Antibody Preparation

Patients who met the current criteria of anti-NMDAR encephalitis were enrolled in this study [28]. Prior to the initiation of immunotherapy, cerebrospinal fluid (CSF) samples were collected with informed consent. CSF samples from patients with non-inflammatory central nervous system disorders were used as controls. The collection of human samples was approved by the Institutional Review Board of Children’s Hospital of Chongqing Medical University. The CSF samples were pooled and purified using Protein A agarose (P2006, Beyotime, Shanghai, China). Following dialysis against phosphate-buffered saline (PBS), the patients’ CSF was concentrated using Amicon Ultra centrifugal filters (UFC5030, Sigma-Aldrich, St. Louis, MO, USA) with a final concentration of 2 mg/dL [25]. Subsequently, the pre-treated CSF samples were confirmed for the presence of anti-NMDAR antibodies through an in-house cell-based assay (CBA), and stored at −80 °C for future analysis. The absence of additional antibodies was confirmed by the loss of specific reactivity of pooled patients’ CSF with rat hippocampal tissue following immunoabsorption using HEK293T cells expressing GluN1, as previously described [10]. Detailed procedures for the CBA and immunostaining are provided in the Supplementary Materials.

### 2.2. Animals

Male C57BL/6J mice (6–8 weeks, 20–24 g) were purchased from the experimental animal center of Chongqing Medical University. All the mice were housed in a controlled environment (12/12 h light/dark cycle, humidity 50–60%, room temperature 22–24 °C) with food and water ad libitum. This study was approved by the Ethics Committees of the Children’s Hospital of Chongqing Medical University (CHCMU-IACUC20220804002).

### 2.3. Stereotactic Intracerebroventricular Injection

The stereotactic intracerebroventricular injection was performed as previously reported [29]. Briefly, mice were anesthetized with sodium pentobarbital (0.375 mg per 10 g of body weight, with a concentration of 7.5 mg/mL) and placed in a stereotactic frame (RWD, Shenzhen, China). After exposing the skull surface, a Hamilton syringe was inserted at the following coordinates relative to the bregma: 1.0 mm left lateral, 0.4 mm posterior, and 2.0 mm depth; this was validated by injecting methylene blue prior to the experiment (Supplementary Figure S1). Eight microliters of purified anti-NMDAR antibodies or controls were slowly pumped (KD Scientific Inc., Holliston, MA, USA) into the lateral cerebral ventricle over 10 min, and the syringe was kept in place for 5 min to enable CSF to diffuse sufficiently [30]. Mice were allowed to recover in a warm chamber and transferred back to their cages.

### 2.4. Study Design and Drug Administration

**Figure 1:**
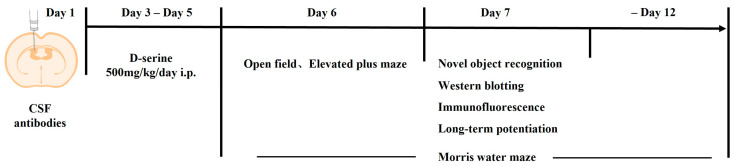
The experimental procedure. CSF: cerebrospinal fluid; i.c.v: intracerebroventricular injection; i.p: intraperitoneal injection.

Figure 1 presents the experimental procedure. One hundred and four mice were randomly allocated into four groups. The sample size was determined based on previous experiences and the 3-R principle. We noticed that a prior study initially assessed the seizure threshold 48 h following intracerebroventricular injection of anti-NMDAR antibodies [30]. Concurrently, another study has demonstrated that, within 2 days post-injection of anti-NMDAR antibodies, a notable reduction in hippocampal NMDAR1 protein levels was already evident in mice [26]. Building upon these findings, we commenced intervention 48 h post-surgery through intraperitoneal injection of D-serine (500 mg/kg, S4205, Sigma-Aldrich) for 3 consecutive days. D-serine was dissolved in 0.9% sterile saline, and the dosage selected was based on previous research with slight modifications [31]. An equal volume of sterile saline was given as a blank control. To reduce the use of animals, mice that underwent open field, elevated plus maze, and novel object recognition tests were sacrificed on that day, and their brain samples were used for Western blotting and immunofluorescence staining.

### 2.5. Behavioral Tasks

The open field (OF), elevated plus maze (EPM), novel object recognition (NOR), and Morris water maze (MWM) tests were conducted to assess symptoms related to anti-NMDAR encephalitis. All tasks were performed by researchers who were blind to the group assignment at the same time. The behavioral parameters were recorded using the ANY-maze tracking system (Stoelting, Chicago, IL, USA).

Briefly, for the OF test, mice were placed in the center of a box and allowed to explore freely for 5 min. The total distance traveled, number of entries to the center, and time spent in the center were recorded. In the EPM test, mice were placed on the central platform facing the open arms and allowed to explore the maze for 5 min. The number of entries and time spent in the open and closed arms were recorded. In the NOR test, the mice explored two identical objects for 5 min, then returned to their home cage. After a 4 h retention interval, one object was replaced with a novel object, and the mice were allowed to explore for 5 min. The recognition index = (time spent on the novel object/total time spent on both objects) × 100%. Detailed procedures are provided in the Supplementary Materials.

### 2.6. Electrophysiological Study

Details of brain slice processing and extracellular field excitatory postsynaptic potential (fEPSP) evocation are provided in the Supplementary Materials. After recording the baseline fEPSPs for 30 min, LTP was induced at the CA3-CA1 synapses by high-frequency stimulation (100 Hz, 1 s duration). The potentiated fEPSPs were recorded at 28 s intervals for 60 min. LTP was quantified by calculating the percentage change in the potentiated fEPSP slope to the average baseline fEPSP slope over the last twenty minutes. The recordings were analyzed using Clampfit 10.7 software.

### 2.7. Immunofluorescent Staining

Seven micrometers of hippocampal coronal sections were prepared using a freezing microtome (Leica, Wetzlar, Germany). The sections were blocked with 5% goat serum containing 0.3% Triton X-100 (T8787, Sigma-Aldrich) at room temperature (RT) for 1 h, and then incubated with mouse monoclonal anti-NMDAR antibodies (67717-1-Ig, 1:500, Proteintech, Wuhan, China) at 4 °C overnight, goat anti-mouse Alexa Fluor 555 (A-21422, 1:2000, Invitrogen, Carlsbad, CA, USA) at RT for 1 h, rabbit polyclonal anti-PSD-95 antibodies (ab18258, 1:500, Abcam, Cambridge, UK) at 4 °C overnight, goat anti-rabbit Alexa Fluor 488 (A-11008, 1:2000, Invitrogen) at RT for 1 h, and DAPI (DA001, LEAGENE, Anhui, China) at RT for 15 min in sequence. Slides were then mounted with ProLong Gold antifade reagent (P10144, Invitrogen) and scanned using a confocal microscope (Nikon C2+, Tokyo, Japan). NMDAR and PSD-95 cluster densities were analyzed using Image J software (v1.54f). One area from each of the hippocampus regions CA1, CA3, and DG was analyzed to calculate the average hippocampal fluorescence density. The average fluorescence density of mice in the normal control group was set as a reference, and the relative fluorescence densities of each group were counted.

### 2.8. Western Blotting

The membrane proteins were extracted from the hippocampus (KGP350, KeyGEN BioTECH, Nanjing, China) and their concentration was determined using the BCA assay (23225, Thermo Scientific, Waltham, MA, USA). Thirty micrograms of each sample were then separated by SDS-polyacrylamide gel electrophoresis and the proteins were transferred onto polyvinylidene difluoride membranes. The membranes were then blocked with 5% skim milk at RT for 1 h. The stripes were then incubated with rabbit monoclonal NMDAR1 antibodies (ab109182, 1:4000, Abcam, Cambridge, MA, USA), mouse monoclonal anti-EphB2 antibodies (sc-130068, 1:500, Santa Cruz, CA, USA), and mouse monoclonal anti-GAPDH antibodies (60004-1-Ig, 1:10,000, proteintech) at 4 °C overnight. After washing, the membranes were incubated with the corresponding secondary antibodies (511103, 511203, 1:5000, Zenbio, Chengdu, China) for 1 h at RT. Finally, the target proteins were visualized using electrochemiluminescence substrate (Bio-Rad, Hercules, CA, USA). Normalized protein expression was analyzed using ImageLab 6.0 software (Bio-Rad).

### 2.9. Statistical Analysis

The data are presented as the mean ± standard deviation (SD). To assess normality, the Shapiro–Wilk test was performed with an α-error of 0.5. Depending on the data distribution, either a one-way or two-way ANOVA was selected to determine differences among groups. Multiple comparisons were conducted using Tukey’s test. All statistical analyses were performed using GraphPad Prism version 9 (GraphPad Software, La Jolla, CA, USA). A *p* value less than 0.05 was considered statistically significant.

## 3. Results

### 3.1. Purification of Patients’ Antibodies

**Figure 2:**
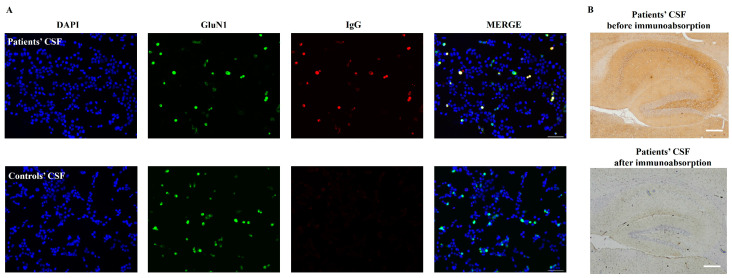
(**A**) Detection of anti-NMDAR antibodies in purified CSF using cell-based assays. Scale bar = 100 μm. (**B**) Immunostaining of sagittal rat brain sections with pooled patients’ CSF before (upper panel) and after (lower panel) immunoabsorption using HEK293T cells expressing GluN1. Scale bar = 200 μm.

CSF samples from three patients diagnosed with anti-NMDAR encephalitis were collected during regular clinical practice. All patients’ CSF tested positive for anti-NMDAR antibodies, but negative for anti-AMPA1, -AMPA2, -LGI1, -CASPR2, -GlyR1, -GABAA, -GABAB, -IgLON5, -DPPX, -DRD2, -GAD65, -mGluR1, -mGluR5, -Neurexin-3α, -AChR, -KLHL11, -GluK2, -AK5, -AGO, -CaVα2δ, -AQP4, -MOG, and -GFAP antibodies by commercial CBA (Kingmed Diagnostics, China). CSF samples from three patients with non-inflammatory central nerve system disorders were also collected as controls. Table 1 provides an overview of their basic clinical information. Prior to conducting animal procedures, the purified anti-NMDAR antibodies from the patients’ CSF samples were confirmed (Figure 2).

### 3.2. A Single Injection of Patients’ Antibodies Does Not Induce Behavioral Changes Associated with Anti-NMDAR Encephalitis in Mice

**Figure 3:**
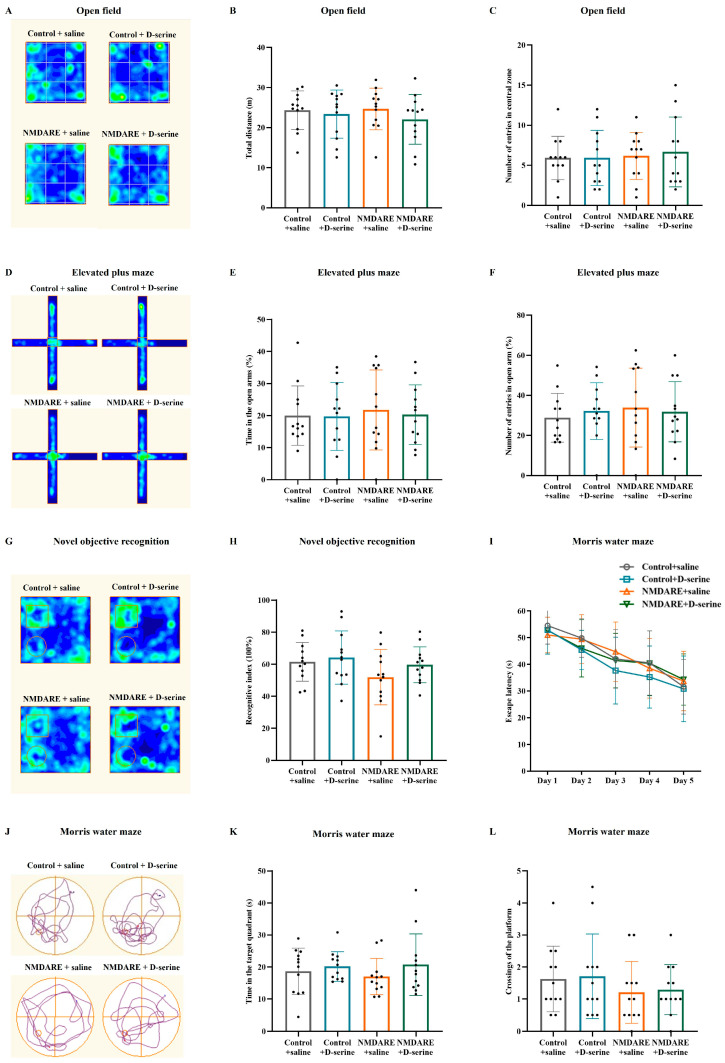
Evaluation of anti-NMDAR encephalitis-related behaviors. (**A**–**C**) Open field test, (**D**–**F**) elevated plus maze test, (**G**,**H**) novel objection recognition, (**I**–**L**) Morris water maze. N = 12 mice in each group. Analysis was performed using two-way ANOVA and post hoc analysis with Tukey’s multiple comparisons test.

Three days after surgery, a series of behavioral tests were conducted to evaluate anxiety or cognitive function in mice. As shown in Figure 3, no significant differences were observed in total distance (24.66 vs. 23.34 m, *p* = 0.9968, Figure 3B) or number of crossings of the central zone (6.17 vs. 5.92, *p* = 0.9971, Figure 3C) in OF between anti-NMDAR encephalitis mice and control mice. Mice injected with anti-NMDAR antibodies and controls showed similar times spent in the open arms (21.74% vs. 19.99%, *p* = 0.9566, Figure 3E) and the number of open arm entries (33.88% vs. 28.83%, *p* = 0.6418, Figure 3F) in EPM. In NOR, mice injected with anti-NMDAR antibodies only exhibited a mild reduction in recognition index compared to the controls, without significant differences (51.90% vs. 61.48%, *p* = 0.1472, Figure 3H). In MWM, all mice showed a similar trend of a decrease in escape latency (*p* = 0.7736, Figure 3I), and mice injected with anti-NMDAR antibodies also exhibited a similar time spent in the targeted quadrant (17.02 vs. 18.69, *p* = 0.7740, Figure 3K) and a similar number of crossings of the platform (1.21 vs. 1.63, *p* = 0.5994, Figure 3L) when compared with the controls. In addition, mice treated with D-serine presented no substantial changes in all tests compared to blank controls, and only a slight recovery of recognition index was observed in anti-NMDAR encephalitis mice treated with D-serine compared to saline controls (59.65% vs. 51.90%, *p* = 0.2629, Figure 3H). These results suggest that a single injection of patients’ anti-NMDAR antibodies into the lateral cerebral ventricle would not induce anxiety-like symptoms or cognitive impairment in mice. Meanwhile, exogenous application of D-serine did not change the mice’s behaviors.

### 3.3. D-Serine Improved Anti-NMDAR Antibody-Mediated Hippocampal LTP Impairment in Mice

**Figure 4:**
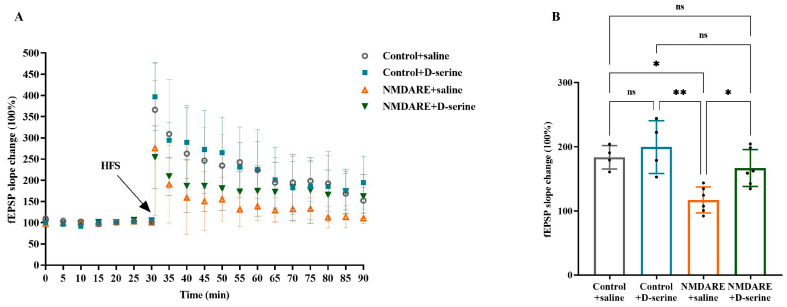
Assessment of hippocampal long-term potentiation. (**A**) Traces of fEPSP recording. (**B**) Quantification of fEPSP changes during the last 20 min. N = 4 recordings from 3 mice for the control + saline and control + D-serine groups; N = 6 recordings from 5 mice for the NMDARE + saline group; N = 6 recordings from 4 mice for the NMDARE + D-serine group. Analysis was performed using two-way ANOVA and post hoc analysis with Tukey’s multiple comparisons test. * *p* < 0.05; ** *p* < 0.005; ns: no significance. HFS: high-frequency stimulation.

Hippocampal synaptic plasticity was assessed by investigating LTP. As shown in Figure 4, mice injected with anti-NMDAR antibodies displayed a notable reduction in the fEPSP slope trace (Figure 4A) and a significant decrease in the mean fEPSP change in the quantitative analysis (117.17% vs. 183.74%, *p* = 0.0194, Figure 4B) compared to control mice. Notably, anti-NMDAR encephalitis mice treated with D-serine, but not saline, exhibited a significant improvement in LTP (166.91% vs. 117.17%, *p* = 0.0392, Figure 4B). These data suggest that D-serine treatment can alleviate the impairment of synaptic plasticity caused by patients’ anti-NMDAR antibodies.

### 3.4. D-Serine Treatment Could Not Reverse Hippocampal NMDAR1 Expression

**Figure 5:**
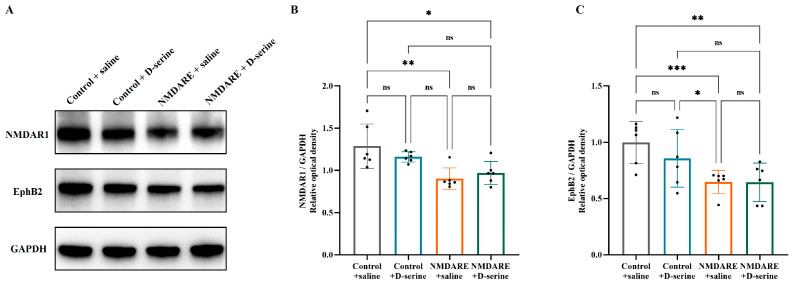
Detection of hippocampal NMDAR1 and EphB2 protein levels in membrane. (**A**) Representative images of Western blotting. (**B**) Quantification of NMDAR1 protein. (**C**) Quantification of EphB2 protein. N = 6 mice per group. Analysis was performed using two-way ANOVA and post hoc analysis with Tukey’s multiple comparisons test. * *p* < 0.05; ** *p* < 0.005; *** *p* < 0.001; ns: no significance.

We further evaluated whether D-serine treatment could improve pathological changes mediated by anti-NMDAR antibodies at the molecular level. The results of Western blotting found that the relative levels of membrane NMDAR1 (0.90 vs. 1.29, *p* = 0.0044, Figure 5A,B) and EphB2 (0.65 vs. 1.00, *p* = 0.0006, Figure 5A,C) proteins in the hippocampus of mice injected with patients’ anti-NMDAR antibodies were significantly lower than those of control mice. However, treatment with D-serine did not alter the membrane levels of NMDAR1 (0.97 vs. 0.90, *p* = 0.8837, Figure 5A,B) or EphB2 (0.64 vs. 0.65, *p* = 0.7343, Figure 5A,C) proteins in anti-NMDAR encephalitis mice. Additionally, control mice treated with D-serine presented a mild decrease in both NMDAR1 (1.16 vs. 1.29, *p* = 0.5383) and EphB2 (0.86 vs. 1.00, *p* = 0.1862) expressions.

**Figure 6:**
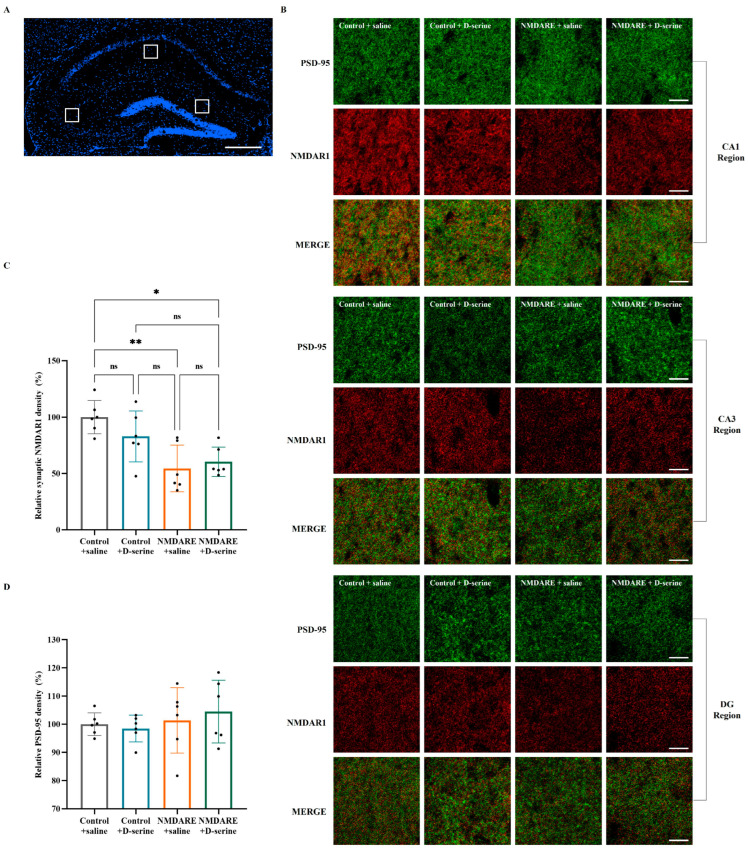
Detection of hippocampal postsynaptic NMDAR density. (**A**) Each area from the CA1, CA3, and DG regions was analyzed as indicated by the white box; scale bar = 500 μm. (**B**) Representative immunofluorescence images of hippocampal CA1 regions: red: NMDAR1; green: PSD-95; merge: postsynaptic NMDAR; scale bar = 10 μm. (**C**) Quantification of relative postsynaptic NMDAR density. (**D**) Quantification of relative PSD-95 density. N = 6 per group. Analysis was performed using two-way ANOVA and post hoc analysis with Tukey’s multiple comparisons test. * *p* < 0.05; ** *p* < 0.005; ns: no significance.

The classical synaptic marker PSD-95 was utilized to label synaptic NMDAR during immunofluorescence. One area selected from the CA3, CA1, and DG regions was used to calculate the mean density, reflecting the overall hippocampal level (Figure 6A). Representative images of synaptic NMDAR1 clusters from each region are shown in Figure 6B. Compared with the controls, mice injected with anti-NMDAR antibodies exhibited a significant reduction in synaptic NMDAR cluster density in the hippocampus (54.44% vs. 100.00%, *p* = 0.0062, Figure 6C). However, treatment with D-serine only led to a slight, albeit non-significant alteration in NMDAR1 cluster density in mice injected with anti-NMDAR antibodies (60.36% vs. 54.44%, *p* = 0.9546). Meanwhile, control mice treated with D-serine also presented a mild decrease in synaptic NMDAR1 cluster density (82.89% vs. 100.00%, *p* = 0.4696). Furthermore, there were no significant differences in PSD-95 cluster densities among the four groups (*p* = 0.6205, Figure 6D).

## 4. Discussion

As an autoimmune-mediated encephalitis, the pathogenicity of anti-NMDAR antibodies has been well established both in vitro and in vivo [8]. However, standard immunotherapy that can effectively eliminate auto-antibodies and inhibit inflammatory reactions is insufficient in controlling symptoms, especially cognitive impairment, in severe cases [10]. Therefore, developing novel targets or treatment strategies is an urgent field to focus on. The results of the current study suggest for the first time that exogenous application of D-serine could improve hippocampal synaptic plasticity in mice intracerebroventricularly injected with patients’ anti-NMDAR antibodies, which could serve as a potential alternative for treating cognitive impairment in patients with anti-NMDAR encephalitis.

Hippocampal synaptic plasticity plays a fundamental role in cognitive function, with NMDAR being crucial for maintaining its structure and function. The activation of NMDAR not only requires the binding of glutamate, but also the co-agonist D-serine. Only when glutamate and D-serine bind to the NMDAR simultaneously can NMDAR be activated to conduct its physiological function [32]. Therefore, the exogenous supplementation of D-serine has been explored as a potential treatment to facilitate cognitive function by improving NMDAR hypofunction and synaptic plasticity in multiple animal models of neurological disease and clinical trials [15,16,17,18,19,20,21,22]. Previous studies have also discussed the possibility of positively modulating NMDAR function in anti-NMDAR encephalitis. Among these, SGE-301, a positive allosteric modulator of NMDAR, was first shown to restore NMDAR hypofunction induced by anti-NMDAR antibodies in vitro [9] and improve hippocampal synaptic plasticity in vivo [10,11]. Similarly, pregnenolone sulfate, a neurosteroid and positive allosteric modulator of NMDAR, has been demonstrated to upregulate NMDAR function and reduce ictal seizure activity in ex vivo brain slices treated with patients’ anti-NMDAR antibodies [33]. Our results also indicated that applying an NMDAR co-agonist could improve anti-NMDAR antibody-induced LTP impairment in mice. Taken together, these studies suggested that NMDAR hypofunction is an important mechanism for cognitive impairment in patients with anti-NMDAR encephalitis, and positive NMDAR modulators, such as D-serine, could serve as a complementary treatment to immunotherapy in the future.

A reliable animal model is crucial for investigating the pathophysiology of and novel treatments for anti-NMDAR encephalitis. However, in this study, mice with a single intracerebroventricular injection of anti-NMDAR antibodies did not display cognitive impairment despite showing significant impairment in hippocampal LTP. It has been demonstrated in other studies that continuous intracerebroventricular infusion of patients’ anti-NMDAR antibodies for 14 days using osmotic pumps significantly impairs cognitive function in mice [25]. This suggests that the dosage of the antibodies from a single injection may be insufficient. Similar results were reported by Wright’s and Taraschenko’s groups, where mice with a single injection of patients’ anti-NMDAR antibodies into the lateral cerebral ventricle only showed a decrease in seizure threshold rather than spontaneous seizures [30]. In contrast, a mouse model of seizures was successfully developed by continuous infusion of anti-NMDAR antibodies for two weeks [34]. Several studies have observed cognitive impairment in rodent models by directly injecting patients’ CSF into the hippocampus [26,27]. This model could be used to explore the pathogenicity of anti-NMDAR antibodies. However, acute hippocampus injury due to the operation makes it less valuable for assessing cognitive function after intervention. In general, despite the large usage of clinical samples, continuous infusion of patients’ antibodies is the best-suited animal model for studying anti-NMDAR antibody-mediated symptoms. Moreover, previous research has shown that different antibody affinity among patients could be a critical factor in anti-NMDAR antibody pathogenicity [35]. Although the diagnosis is anti-NMDAR encephalitis, the antibodies purified directly from patient serum and cerebrospinal fluid are polyclonal mixtures, which may even include other unknown pathogenic antibodies. As a result, we are unable to definitively quantify or attribute the observed structural and functional changes to a specific anti-NMDAR antibody. Therefore, if available, passive transfer of patient-derived monoclonal anti-NMDAR antibodies is the most reliable scheme for in vivo studies, which calls for much higher technical reserves [36].

The major limitation of this study was its inability to induce behavioral changes related to anti-NMDAR encephalitis in mice. Although electrophysiological studies suggested that D-serine could ameliorate synaptic plasticity, further research is required to determine whether it can alleviate cognitive impairment caused by anti-NMDAR antibodies. In particular, future studies should employ a well-established, continuous infusion model to better mimic the pathological conditions and assess the therapeutic potential of D-serine in this context.

Another critical limitation is that D-serine intervention failed to reverse the downregulation of NMDAR induced by anti-NMDAR antibodies. NMDAR activation triggers downstream signaling pathways such as CaMK, ERK, CREB, and BDNF, which are pivotal in regulating synaptic structure and function [37]. While current studies mainly focus on the direct effects of antibodies on NMDAR, this underscores the need for further investigation into the alterations in downstream signaling pathways mediated by NMDAR in this disease. Given their essential roles in maintaining synaptic plasticity, understanding these pathways could provide valuable insights into the mechanisms underlying the persistent cognitive impairment associated with anti-NMDAR encephalitis.

Additionally, this study did not measure the endogenous levels or dynamic changes in D-serine in anti-NMDAR encephalitis mice. In patients with schizophrenia, decreased serum or CSF D-serine levels have been associated with cognitive impairment [38]. Considering the potential parallels between these conditions [23], it is worth investigating whether changes in D-serine levels have similar impacts in anti-NMDAR encephalitis.

## 5. Conclusions

In conclusion, our results emphasize the potential of D-serine application in improving hippocampal synaptic plasticity impairment in mice due to anti-NMDAR antibodies. Additional research is required to determine whether D-serine treatment can further alleviate cognitive impairment, and how exogenous D-serine impacts hippocampal synaptic plasticity and cognitive function in a well-established animal model of anti-NMDAR encephalitis.

## Figures and Tables

**Table 1:** Clinical information of cerebrospinal fluid samples used in this study.

Samples	Age	Gender	Diagnosis	Abs Titer
Patient 1	12 years 3 months	Male	Anti-NMDAR encephalitis	1:100
Patient 2	6 years 8 months	Female	Anti-NMDAR encephalitis	1:32
Patient 3	7 years 11 months	Female	Anti-NMDAR encephalitis	1:32
Control 1	10 years 10 months	Male	Migraine	-
Control 2	8 years 7 months	Female	Benign intracranial hypertension	-
Control 3	5 years 9 months	Female	Migraine	-

NMDAR: N-methyl-D-aspartate receptor, Abs: antibodies.

## Data Availability

All data are available on reasonable request from the corresponding authors.

## Supplementary Materials

The following supporting information can be downloaded at https://www.mdpi.com/article/10.3390/biomedicines12122882/s1. Figure S1: Validation of the coordinates by intracerebroventricularly injecting methylene blue. Red arrow: methylene blue was diffused in both ventricles of mice.
