# Supplementary material for: D-Serine May Ameliorate Hippocampal Synaptic Plasticity Impairment Induced by Patients’ Anti-N-methyl-D-aspartate Receptor Antibodies in Mice
Source: Biomedicines. 2024 Dec 18;12(12):2882. doi: 10.3390/biomedicines12122882 (PMC11673065; doi:10.3390/biomedicines12122882)
Supplement: Supplementary file 1 [file biomedicines-12-02882-s001.zip › biomedicines-3308754-supplementary.pdf]

Supplementary Materials

D-serine may ameliorate hippocampal synaptic plasticity impairment induced by patients' anti-N-methyl-D-aspartate receptor antibodies in mice

Supplementary Methods

Cell-based assay

Human embryonic kidney 293 (HEK293) cells were transfected with full-length GFP-tagged NR1 and NR2B subunits of the NMDAR. After 24 hours of incubation, the cells were fixed with 4% paraformaldehyde for 15 minutes. The cells were then blocked and permeabilized in a solution containing 5% bovine serum albumin (BSA) and 0.3% Triton™ X-100 for 1 hour. Purified CSF samples were incubated with the transfected cells overnight at 4°C. On the following day, bound antibodies were detected using Alexa Fluor 555-conjugated goat anti-human IgG (1:2000, A-21445, Thermo Fisher Scientific) for 1 hour at room temperature (RT). The nuclei were counterstained with DAPI (DA0001, LEAGENE, China) at RT for 15 minutes. Finally, the slides were mounted with ProLong Gold antifade reagent (P10144, Invitrogen) and visualized under a Nikon 90i fluorescence microscope (Nikon, Japan).

Immunohistochemistry

Seven-micrometer frozen sagittal sections of rat brains were prepared for immunostaining. Pooled patients' CSF, before and after immunoabsorption, was applied to the sections and incubated overnight at 4°C. Immunoperoxidase staining was performed using a commercial SABC-POD (Human IgG) Detection Kit (SA1024, Boster Biological Technology, China), following the manufacturer's instructions. After 3,3'-diaminobenzidine (DAB, AR1022, Boster) staining, the sections were lightly counterstained with hematoxylin (AR0005, Boster) and mounted with neutral balsam. The slides were then visualized and photographed using a Nikon Eclipse Ts2R microscope (Nikon, Japan).

Open field test.

Each mouse was placed in the center of a plastic box (40cm x 40cm x 30cm) and allowed to explore freely for 5 minutes. The total distance traveled, the number of

crossings to the central area, and the time spent in the center area were then recorded.

Elevated plus maze test.

The Elevated plus maze was composed of two closed arms (5 × 35 × 15 cm), two open arms (5 × 35 cm), and a central platform (10 × 10 cm). Each mouse was positioned on the central platform with their heads facing the open arms and was allowed to remain in the maze for 5 minutes. The number of entries and the time spent in the open and closed arms were then recorded.

Novel object recognition.

The novel object recognition test was performed using the same box as the open field test. Firstly, mice were placed into the box without any objects for a 5-minute adaptation period. On the following day, the mice were placed in the box to explore two identical objects for 5 minutes, and then returned to their home cage. After a 4 h retention interval, the animals were allowed to explore both objects again for 5 minutes. In the box, they were presented with two different objects, one of which was identical to the object they had encountered earlier, and the other was a novel object. The two objects were fixed to the bottom of the box, with their positions counterbalanced between animals, at least 10 cm away from the walls of the box. A mouse was considered to be exploring an object when it was facing the object with its nose within 2 cm of the object. The time each animal spent actively exploring the objects was recorded. The recognition index = time spent on the novel object / total time spent on both objects x 100%.

Morris water maze test.

The MWM apparatus consisted of a circular pool (150 cm diameter) filled with warm water (25 ± 1°C). The pool was divided into four quadrants using the four cardinal points (N, E, S, W). Different visual objects were hung as spatial cues. On the first day, each mouse was introduced to the pool for 60 seconds to familiarize themselves with the maze. From the second day to the sixth day, a round platform (10cm diameter) was submerged in the middle of the northwest quadrant (Target quadrant). Mice were placed into the pool at each of the four quadrants and were allowed to swim freely until they located the hidden platform, where they remained for 3 seconds. Escape latency was

defined as the time taken for the mice to locate the hidden platform. If mice failed to find the platform within 1 minute, they were guided to it and remained there for 20 seconds. The escape latency was recorded as 60 seconds. On the seventh day, the platform was removed, and mice were placed in the pool for 60 seconds. The number of crossings over the platform location and the time spent in the target quadrant were recorded.

Neurophysiological tests

Brain slices preparation.

The mice were sacrificed following anesthesia with isoflurane. The brain was rapidly removed and submerged in an ice-cold cutting solution containing (in mM) 3 KCl, 1.25 NaH2PO4, 26 NaHCO3, 0.4 ascorbic acid, 2 pyruvate-Na, 2 lactate-Na, 10 D-glucose, 2 MgCl2, 4 MgSO4, 0.1 CaCl2 and 220 sucrose, bubbled with a mixture of 95% O2 and 5% CO2. Coronal hippocampal slices (400μm) were prepared using a vibratome (VT1200s, Leica, Germany). The slices were then incubated in oxygenated (95% O2 and 5% CO2) artificial CSF (aCSF) containing (in mM) 124 NaCl, 3 KCl, 1.25 NaH2PO4, 26 NaHCO3, 0.4 ascorbic acid, 2 pyruvate-Na, 2 lactate-Na, 10 D-glucose, 1.2 MgSO4 and 2 CaCl2 at 32°C for 1 hour and then recovery at 22-24°C for at least 1 hour.

Long-term potentiation (LTP) recording.

Hippocampal slices were transferred to the recording chamber with continuous perfusion of oxygenated (95% O2 and 5% CO2) artificial cerebrospinal fluid (aCSF) at a flow rate of 1 mL/min. Extracellular field excitatory postsynaptic potentials (fEPSPs) were evoked from the CA1 stratum radiatum of the hippocampus using a glass pipette filled with aCSF (2–4 MΩ). Evoked responses were amplified using an Axon Multiclamp 700 amplifier (Axon, USA). The recordings were analyzed using Clampfit 10.7 software. After recording baseline fEPSPs for 30 minutes, long-term potentiation (LTP) was induced by high-frequency stimulation (HFS, 100 Hz, 1 s duration). Potentiated fEPSPs were recorded at 28 s intervals for 60 minutes. LTP was quantified by calculating the percentage change of the potentiated fEPSPs slope to the average baseline fEPSPs slope in the last twenty minutes.

Supplementary Figure

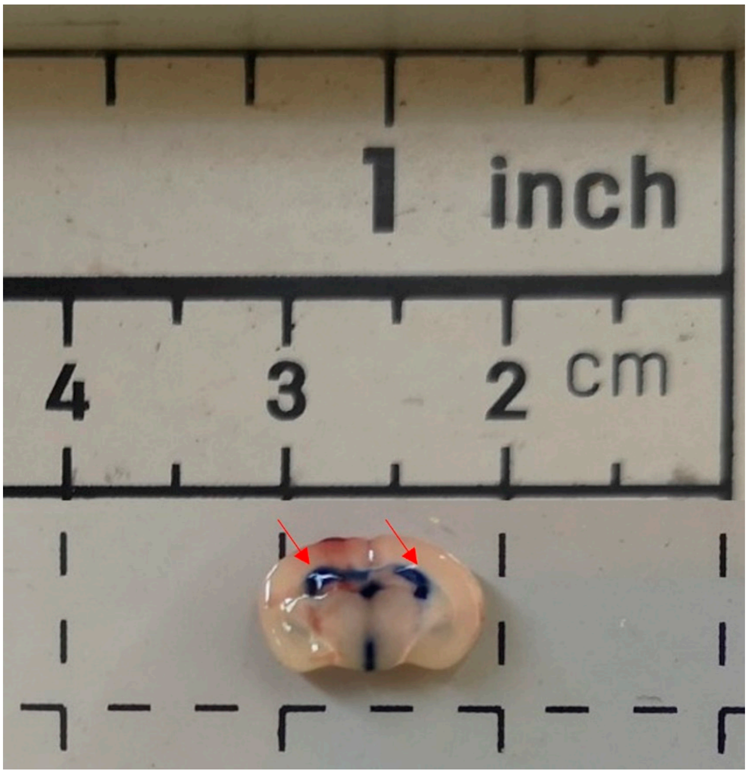

Figure S1 Validation of the coordinates by intracerebroventricularly injecting methylene blue. Red arrow: methylene blue was diffused in both ventricles of mice.
